# The influence of menstrual cycle on metabolic control and diet in patients with phenylketonuria

**DOI:** 10.1186/s13023-025-03890-2

**Published:** 2025-07-13

**Authors:** Laura Horka, Kristin Landolt, Zoran Erlic, Nicole Rimann, Alessio Cremonesi, Michel Hochuli, Felix Beuschlein

**Affiliations:** 1 Clinic for Endocrinology, Diabetology and clinical Nutrition, University Hospital of Zurich, Zürich, Switzerland; 2 Department of Nursing and MTTB (medical-technical and medical-therapeutic professions), University Hospital Zurich, Zürich, Switzerland; 3 Swiss Newborn Screening Laboratory, University Children’s Hospital Zurich, University of Zurich, Zürich, Switzerland; 4 Division of Clinical Chemistry and Biochemistry, University Children’s Hospital Zurich, University of Zurich, Zürich, Switzerland; 5 Department of Diabetes, Endocrinology, Nutritional Medicine and Metabolism, University Hospital Bern, Inselspital, Bern, Switzerland; 6 Medizinische Klinik und Poliklinik IV, Klinikum der Universität, Ludwig-Maximilians-Universität, Munich, Germany; 7 The LOOP Zurich - Medical Research Center, Zurich, Switzerland

**Keywords:** Phenylketonuria, Menstrual cycle, Nutrition therapy, Phenylalanine, Cycle dependent metabolic changes

## Abstract

Based on experiences from everyday clinical practice and indications from the literature, the menstrual cycle might impact on metabolic stability in patients with urea cycle disorders and organic acidurias. However, this connection has not yet been systematically investigated.

Phenylketonuria (PKU) as the most prevalent inborn error of metabolism with its easily determinable biomarker is a suitable model disease to shed light on this question. In ten patients with classic PKU on a low protein diet and on an amino acid mixture, phenylalanine (Phe) was measured from dried blood spots twice a week for 6 months. During this time, the patients documented their menstrual cycle and filled in nutrition protocols since it is known that the menstrual cycle also influences nutritional behavior. Based on this cohort, we found a significant correlation between the phases of the menstrual cycle and Phe concentration, with the lowest concentrations in the early luteal phase and the highest in the early follicular phase, during menstrual bleeding. This effect did not appear to be due to a change in eating behavior, as both protein and calorie intake were not significantly different in relation to the menstrual cycle. Since the increase in Phe began before menstrual bleeding, it does also not appear to be a pure effect of catabolism due to bleeding. Further studies will be required to identify the cause of this effect and to develop possible therapeutic strategies.

## Background

With certain intoxication type inborn errors of metabolism (organic acidurias and urea cycle disorders), a deterioration of metabolic stability in association with menstrual bleeding has been observed in clinical practice. Likewise, there are some case reports in the literature, describing a catamenial deterioration including an increase in ammonia levels in patients with urea cycle disorders [1–4]. Different mechanisms have been postulated as potential causes for the deterioration, including protein overload due to endometrial exfoliation, catabolism during menstrual bleeding, a cycle dependent change in diet or a direct hormonal effect.

It is known that women’s eating behavior changes during the different phases of the menstrual cycle. Various studies have documented an increase in calorie intake, especially through carbohydrates, in the late luteal phase (shortly before menstrual bleeding). Protein intake and diet related cravings also increased during the luteal phase. The resting metabolic rate appeared to be higher in the luteal phase than in the follicular phase, too [5–8].

As the number of patients with both, organic acidurias and urea cycle disorders are very small and metabolic control in those is much more complex to assess, patients with phenylketonuria (PKU) represent a more suitable and amenable population for a systematic first investigation of this topic. Similar mechanisms leading to metabolic control deterioration during menstrual bleeding are also plausible in PKU patients and knowing about them would be relevant for the affected patients in order to improve their therapy and potentially their outcome.

PKU is the most prevalent inborn error of metabolism with an incidence of estimated 1:10’000 births. It is caused by an autosomal recessively inherited deficiency of the enzyme phenylalanine hydroxylase. Due to this enzyme deficiency, phenylalanine (Phe) from protein breakdown cannot be metabolized and accumulates. The high Phe concentrations have a negative impact on neurological development, especially at a young age, with severe disabilities in case of insufficient treatment [9]. In many countries, PKU is screened in newborns, in order to identify those patients as early as possible to initiate therapy and thus enable a normal development of affected children. Depending on the severity of the enzyme deficiency and the Phe concentration at birth, the disease can be classified into classic PKU, mild PKU and mild hyperphenylalaninemia. The standard treatment for patients with classic PKU consists of a severely protein restricted diet (often using low-protein medical foods) and supplementation with a phenylalanine-free amino acid mixture to meet protein requirements. It is currently unknown, how the menstrual cycle affects patients eating habits and metabolic stability in inborn errors of metabolism in general or, more specifically in PKU. Because patients with PKU follow a very strict diet with little variability, it is not a given that observations regarding the cycle-dependent changes in eating behavior of women in the general population will be true for patients with classical PKU as well. Therefore, the aim of this study was to investigate the possible effect of the menstrual cycle on the Phe concentrations as well as on the nutritional habits.

This project is intended to narrow this knowledge gap and improve the basic understanding of this disease in order to achieve a better metabolic control in PKU patients by specific therapy adjustments, which might be transferrable to other metabolic diseases.

## Patients and methods

**Fig. 1:**
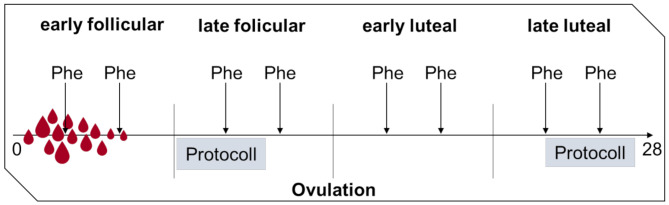
Study design: Phe measurements from dried blood spots twice weekly, nutrition logs over three days during mid follicular and late luteal phase and documentation of menstrual cycle dates

The project was approved by the Ethics committee of canton Zurich. The aim of this study was to investigate whether Phe concentrations are significantly different, depending on the phases of the menstrual cycle and whether this difference can be explained by changes in diet or not. We recruited 10 patients with classic PKU with a regular menstrual cycle and without a planned pregnancy in the near future, willing to participate in this study. Informed consent was obtained from all patients. The inclusion and exclusion criteria are listed in Table 1, the study design is summarized in Fig. 1 and the baseline characteristics are provided in Table 2. Notably, 9 of 10 patients had no hormonal contraception and 1 patient used combined oral contraception.

**Table 1:** Inclusion and exclusion criteria

Inclusion criteria	Exclusion criteria
Classical PKU	Inability to provide informed consent
Female sex	BH4-responsiveness and -treatment
Age ≥ 18 years	Enzyme replacement therapy
Regular menstrual cycle (28 +/- 7 days)	Irregular menstrual cycles (cycle-length > 40 days)
Established therapy by protein-reduced diet and phenylalanine-free amino acid mixture	Planned or current pregnancy, lactation
Willing to conduct frequent Phe measurements and to perform a diet protocol	Continuous hormonal therapy without classical menstrual cycle (e.g. continuous progestin pill or intrauterine device)
	Intensive physical activity (more than 30 min daily in case of high intensity and 4 h daily in case of low intensity activity)

**Table 2:** Baseline characteristics of included patients

	Average	Minimum	Maximum
Age [y] (range)	33.8	23	50
BMI [kg/m2]	24.9	19.1	32.5
Prescribed natural protein (g/d)	9.8	3	15
Prescribed synthetic protein equivalent (g/d)	50.5	38	70
Phe concentration before study (average of last 3 measurements in µmol/l)	758.8	660	886

The patients received a set of pre-labeled, pseudonymized filter papers and envelopes to collect blood for Phe measurement, as well as a lancing device and lancets for the self-sampling of capillary blood. They were instructed to collect blood twice a week for 6 months. Amino acids analysis was performed by means of mass spectrometry in an accredited diagnostic laboratory using routine procedures. Briefly, amino acids were extracted from a dried blood spot (Ø 3.2 mm) using the Neomass AAAC Plus newborn screening kit (Labsystems Diagnostics, Finland). A UHPLC Nexera X2 (Shimadzu, Switzerland) coupled to a 8060 Triple Quadrupole mass spectrometer (Shimadzu, Switzerland) with electrospray ionization was used for the quantitative analysis of phenylalanine. Labsolutions and Neonatal Solutions softwares (Shimadzu, Switzerland) were used for data acquisition and data analysis, respectively.

During the 6-month study period the patients were also asked to document the menstrual cycle using an application on the mobile phone and a paper calendar. And they were instructed to record their diet in detail for 72 h at two different time points for each menstrual cycle (mid follicular and late luteal) using a mobile phone Application (“Mevalia EASY DIET”). The protocols were reviewed and checked by a nutritionist.

Results were entered in a REDCap (Research Electronic Data Capture) database and analyzed with SPSS. To investigate the influence of the menstrual cycle on Phe concentrations, we subdivided the menstrual cycle in four phases: early follicular (EF day 1–7), late follicular (LF day 8– calculated ovulation), early luteal (EL calculated ovulation– 7 days before menstruation) and late luteal (LL 7 days before menstruation– start of menstruation). We then performed a one-way repeated measures ANOVA test with Huynh-Feldt correction for violation of sphericity and a Bonferroni post hoc test. For the changes in diet depending on the menstrual cycle phase we performed paired samples t-tests. The paired samples t-test was also used to calculate the effect of study participation on the Phe concentration. Level of significance was set at two-sided α = 0.05.

## Results

**Fig. 2:**
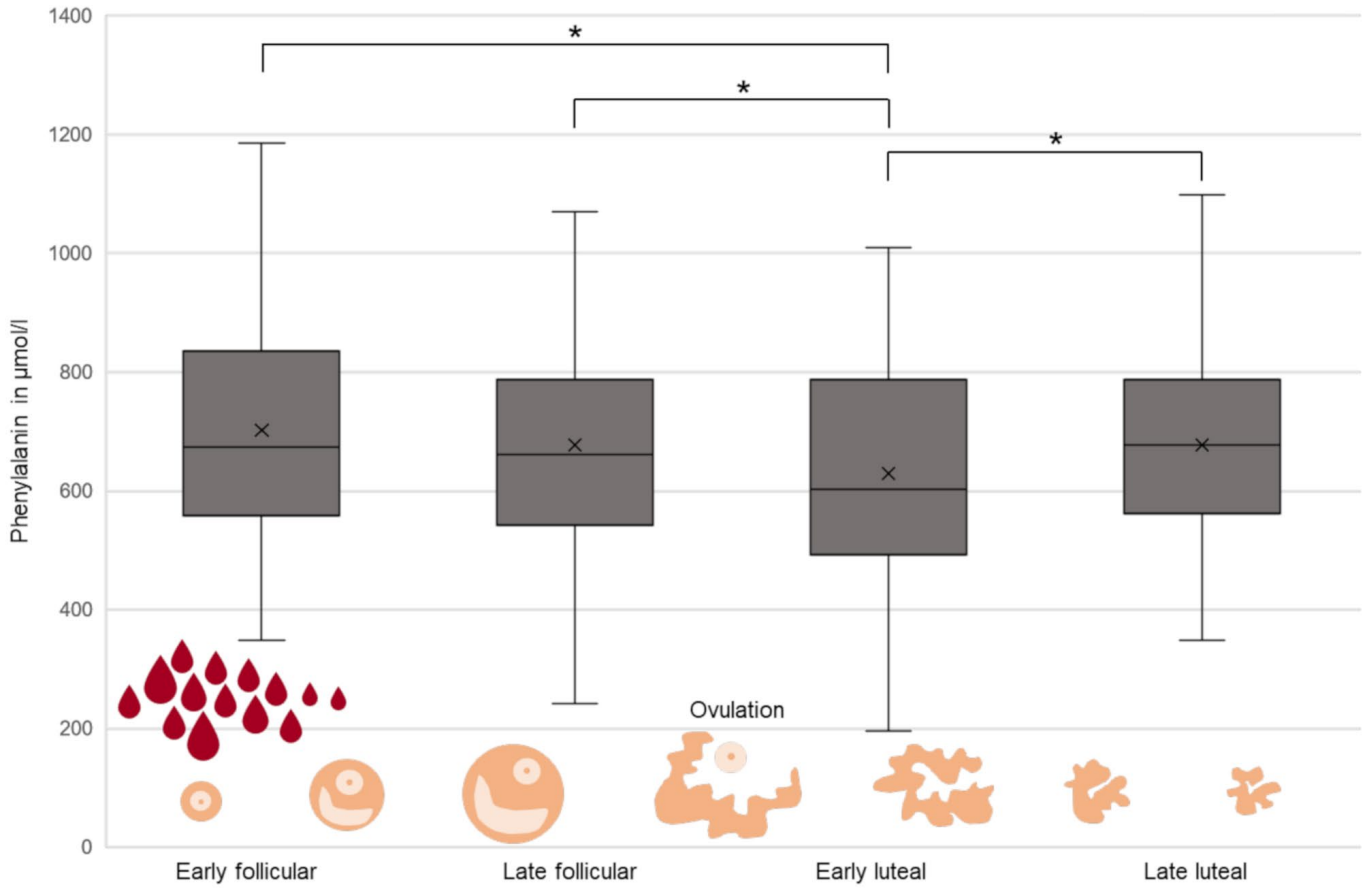
Phe concentrations depending on the menstrual cycle phase, early follicular, late follicular, early luteal, late luteal from left to right

Regarding the main endpoint of the study, the impact of the menstrual cycle phase on the Phe concentrations, we could document the lowest Phe concentrations in the early luteal phase (627.9 ± 179.0 µmol/l) and the highest in the early follicular phase (702.2 ± 188.8 µmol/l), as shown in Fig. 2. The difference between the mean Phe concentrations in relation to the cycle phase reached statistical significance *F*(2.85, 271.13) = 5.79, *p* < 0.001.

Bonferroni post hoc test revealed significantly higher Phe concentrations during early follicular (*p* = 0.002, M_diff_ = 73.26 µmol/l, 95%-CI[20.95, 125.58]), late follicular (*p* = 0.032, M_diff_ = 48.68 µmol/l, 95%-CI[2.74, 94.62]) and late luteal phase (*p* = 0.007, M_diff_ = 48.29 µmol/l, 95%-CI[9.60, 86.99]) than during early luteal phase.

In conclusion, significant differences between the mean Phe concentrations in the different cycle phases were documented with the lowest Phe concentrations in early luteal phase, just shortly after ovulation, which rose to a maximum during menstrual bleeding in early follicular phase.

Blood Phe concentrations were on average slightly above the recommended target range of below 600 µmol/l, they tended to be lower during the study period (671.5 ± 123.8 µmol/l) than the average of the last three Phe measurements before the observation period (758.8 ± 67.6 µmol/l), but this difference did not reach significance (*p* = 0.059).

Protein, phenylalanine and calorie intake was not significantly different between the phases of the menstrual cycle phase (follicular or luteal). However, the results for protein and phenylalanine intake must be viewed with the limitation that there were extreme outliers, and the data was not normally distributed. The results are shown in Table 3. Another limitation for this data is the quality of the nutrition protocols. Indeed, the patients had difficulties keeping nutrition logs, which led to incomplete logs in 4 out of 10 women, some of which were also recorded at the wrong time points (e.g. two protocols for the follicular phase, none in the luteal phase). For 2 patients it was unfortunately not feasible to keep a nutrition log, so that no data is available for 2 out of 10 women.

**Table 3:** Average daily intake of calories, protein and phenylalanine in the follicular and the luteal phase

Cycle phase	follicular	luteal
Caloric intake (kcal/d)	1691.5 ± 412.7	1763.0 ± 554.5
Protein intake (g/d)	12.5 ± 6.2	12.9 ± SD 7.3
Phenylalanine (mg/d)	597.7 ± 265.3	599.3 ± 309.3

## Discussion

We were able to show that the menstrual cycle has a significant influence on the Phe concentrations in patients with classic PKU, despite the relatively small number of patients included. To our knowledge, this is the first study investigating this aspect systematically for any inborn error of metabolism, since up to now only a small number of case reports were published on this topic. The Phe concentrations were the lowest in early luteal phase and rose to a maximum in the early follicular phase. We do not attribute this to a change in eating habits as there were no significant differences in protein, phenylalanine or calorie intake depending on the menstrual cycle phase. However, this statement is limited by the quality of the obtained nutrition logs. Indeed, for the patients, obtaining accurate and complete logs on their nutrition was more difficult than we initially expected.

Notably, a pure effect based on catabolism due to blood loss during menstruation seems unlikely, as the Phe concentrations rose before the onset of bleeding. A more plausible explanation would be the effect of hormonal changes during the menstrual cycle. In fact, the course of the Phe concentration was inverse to the estradiol concentration described for the female cycle. In short, estradiol is low during the follicular phase, ovulation is preceded by a high estradiol peak and in the luteal phase estradiol is higher than in the first half of the cycle but falls towards the end of the cycle [10].

Whilst we did not measure estradiol for this study, the Phe concentration was highest at the beginning of the cycle, that is, during the lowest estradiol concentration. The Phe concentration decreased in the first half of the cycle and was at its lowest after the calculated ovulation. This in turn is shortly after the estradiol peak. Towards the end of the cycle, there was another increase in Phe concentration, which could again correspond to the falling estradiol concentrations in the late luteal phase. The mild anabolic effect of estradiol (well documented on endometrium and bone) would also be in line with this theory [11, 12].

Probably the effect of estradiol is further influenced by changes in nutrition and catabolism during the menstrual cycle. The fact that the maximum Phe concentrations coincided with menstrual bleeding suggests a connection with blood loss, which is also supported by the fact that the same course occurred in the patient on oral contraception. Also, a change in diet could influence Phe concentrations, too but this could not be statistically proven in our study even though it was reported subjectively by many patients.

An interesting observation in our study was an improvement in Phe concentrations documented during the study compared to the last three measured values before the observation period, probably explained by a more careful focus on the diseases by each patient.

The results of our study are clinically relevant, although it needs to be acknowledged, that a maximum difference in Phe of 10.7% is not decisive for the clinical routine of patients. Menstrual-cycle dependent changes of metabolic control may be more relevant for patients with intoxication-type disorders, however, for better feasibility the study was deliberately conducted on patients with PKU as a model system for inborn errors of protein catabolism. It remains to be demonstrated whether the results can be extrapolated to patients with other inborn errors of metabolism such as intoxication type disorders.

Future studies will be necessary to reinforce our findings and define possible intervention options. One approach could be to reduce the natural protein intake in patients with regular menstrual cycles shortly before menstruation and at the same time slightly increase the calorie intake.

It would be particularly interesting to see whether the same effect of the menstrual cycle on metabolic stability is also observed in patients suffering from organic acidurias or urea cycle disorders for example and whether similar intervention strategies could be applied to them to improve metabolic control.

## Conclusion

A significant effect of the menstrual cycle on Phe concentration could be demonstrated with the lowest Phe concentrations shortly after ovulation and highest concentrations in the late luteal phase. The etiology of this effect couldn’t be completely clarified with this current study, possible explanations include catabolism due to blood loss, changes in nutritional habits depending on the cycle phase and a direct hormonal effect.

## Data Availability

The data that support the findings of this study are available from the corresponding author.

## Abbreviations

PKU: Phenylketonuria
Phe: Phenylalanine
REDCap: Research Electronic Data Capture
EF: Early follicular
LF: Late follicular
EL: Early luteal
LL: Late luteal
